# Taxonomy of the thelyphonid genus *Typopeltis* Pocock, 1894, including homology proposals for the male gonopod structures (Arachnida, Thelyphonida, Typopeltinae)

**DOI:** 10.3897/zookeys.848.32263

**Published:** 2019-05-20

**Authors:** Gabriel Seraphim, Alessandro Ponce de Leão Giupponi, Gustavo Silva de Miranda

**Affiliations:** 1 Laboratório de Referência Nacional em Vetores das Riquetsioses, LIRN-IOC-FIOCRUZ. Manguinhos, 21040-360, Rio de Janeiro, RJ, Brazil Laboratório de Referência Nacional em Vetores das Riquetsioses Rio de Janeiro Brazil; 2 Department of Entomology, National Museum of Natural History, Smithsonian Institution, Washington D.C., USA National Museum of Natural History, Smithsonian Institution Washington United States of America

**Keywords:** Asian fauna, taxonomy, Uropygi, Vinegaroons, whip scorpion

## Abstract

The genus *Typopeltis* Pocock, 1894 is poorly known regarding its systematics, natural history, and distribution, despite important taxonomic advances during the 1990s. Currently, only 13 species are known from East Asia, including areas in south China, Japan, Vietnam, Laos, Thailand, and Taiwan. In this work, we describe and illustrate a new species of *Typopeltis* from Vietnam and provide a new description for the male of *T.guangxiensis* Haupt & Song, 1996. Additionally, we describe and illustrate the female gonopod of *T.guangxiensis* for the first time and propose a new homology hypothesis for the male gonopod parts. The male of *T.laurentianus***sp. n.** is characterized by the unique patellar apophysis that presents a smooth texture and no spines. *Typopeltislaurentianus***sp. n.** is the third species of this genus to be described from Vietnam.

## Introduction

The order Thelyphonida Latreille, 1804 (also known as whip scorpions) is a conspicuous, yet small arachnid order with only 124 living species in 15 genera described so far (Zhang 2013, Barrales-Alcalá et al. 2018). Despite not being extremely diverse, the group is quite old and is estimated to have originated around 333 mya in tropical Pangea (Clouse et al. 2017). The oldest Thelyphonida fossil is from 318 mya and currently only seven fossils are known (Dunlop et al. 2008, Wolfe et al. 2016). Recent estimates of time divergence indicate an increase in diversification rates during the Cretaceous (Clouse et al. 2017).

Thelyphonida is currently composed of one family (Thelyphonidae) and four subfamilies: Hypoctoninae Pocock, 1899, Mastigoproctinae Speijer, 1933, Thelyphoninae Lucas, 1973 and Typopeltinae Rowland & Cooke, 1973. The classification history of the order goes back to Pocock (1899), who divided the family Thelyphonidae into two subfamilies, Thelyphoninae and Hypoctoninae (Pocock 1899). The two subfamilies were defined by the presence or absence of a keel between the medial and lateral eyes, respectively. Gravely (1916) set up an organization scheme that would become the current classification of the order, although some of the names were given only afterwards (such as Mastigoproctinae Speijer, 1933 and Typopeltinae Rowland & Cooke, 1973). Gravely (1916) divided Thelyphoninae into three groups, Mastigoproctinae and Typopeltinae, and a new one characterized by a strongly modified patellar process. This last group became the currently defined subfamily Typopeltinae, which includes the single genus *Typopeltis* Pocock, 1894 (Rowland and Cooke 1973).

The genus *Typopeltis* is endemic to Asia, with 13 valid species (including the *nomen dubium T.amurensis* (Tarnani, 1889) from Russia). Six of these species are known from Southeast Asia: two from Vietnam (*T.harmandi* Kraepelin, 1900; *T.soidaoensis* Haupt, 1996), three from southern China (*T.vanoorti* (Speijer, 1936); *T.sinensis* (Butler, 1872); *T.guangxiensis* Haupt & Song, 1996) and one from Laos (*T.magnificus* Haupt, 2004) (Haupt 1996, 2004a, Haupt and Song 1996, Harvey 2003). Several areas in East and Southeast Asia remain unsampled or undersampled, such as Cambodia and Thailand (with no records of *Typopeltis* at all), and Laos (with one record). Not only is there little information regarding the group’s distribution, but the systematics of *Typopeltis* is also still in its infancy. The greatest contributor to the understanding of the genus was Joachim Haupt (Haupt and Song 1996, Haupt 2004a, 2009); however, several details of the morphology of the species were not addressed and continue to be unknown, such as the form of the male gonopod.

The genus *Typopeltis* can be easily recognized by the presence of a marked keel between the lateral and median eyes and by the absence of a suture dividing the abdominal tergites (Rowland and Cooke 1973). The males have a well-developed patellar apophysis and no projection on sternite III (Rowland and Cooke 1973). Females have modifications of the tarsomeres of leg I (antenniform), but according to Gravely (1916) this character can vary depending on the age and reproductive period of the specimen. In addition, the females have clear modifications of sternite II (genital plate) compared to males. The trochanter spines, despite being conspicuous, are not used in the taxonomy of the group because they vary considerably, with differences between the right and left pedipalps of a single individual (Gravely 1916).

Not much is known regarding the phylogenetic relationships of *Typopeltis* species. In a molecular phylogeny of Thelyphonida, Clouse et al. (2017) included only one named species of *Typopeltis*, *T.crucifer*, which was recovered as sister to what was most likely an unnamed species in the same genus from Vietnam. Interestingly, Typopeltinae was recovered as being more closely related to Thelyphoninae and Mastigoproctinae than to Hypoctoninae.

In this work, we aim to contribute to the understanding of the morphological characters of *Typopeltis* by describing and illustrating a new species from Vietnam. In addition, we provide the first description of the male of *T.guangxiensis* Haupt & Song, 1996, provide detailed images of the female of that species, and present a hypothesis of homology of the male gonopod structures based on Giupponi and Kury (2013). Our homology hypothesis is made based on the consistent sister group relationship between Amblypygi and Thelyphonida (e.g. Ballesteros and Sharma 2019). With this, we intend to set the basis for the evaluation of new characters in future morphological phylogenetic studies.

## Material and methods

Specimens were identified based on Rowland and Cooke (1973) and Haupt (1996). The description was adapted from Haupt (1996), Víquez and Armas (2007), Giupponi and Vasconcelos (2008), Villarreal and Giupponi (2009). The descriptions were made with NIKON SMZ745 and LEICA MZ15 stereomicroscopes. Photographs were made with a Leica M205C and Leica Application Suite V. 4.7 software. Scanning electron microscope (SEM) images were produced in a JEOL JSM-6390LV. The map was made with ArcGIS 10.3. All images have been edited with Adobe Photoshop CS6 and Adobe InDesing CS6.

**Acronyms**:

**Fi** = fistula; **GO** = genital operculum; **LaM** = lamina medialis; **LoD** = lobus dorsalis; **LoL1** = lobus lateralis primus; **LoL2** = lobus lateralis secundus; **PI** = processus internus; **Me** = Mensa (**new name**); **Fu** = Fulcrum (**new name**); **RS** = receptaculum seminis; **CCh** = circulus chitinosus (**new name**); **ACh** = arcus chitinosus.

**MNRJ**Museu Nacional, Rio de Janeiro, Brazil (the thelyphonid specimens were on loan from the collection when the Museum burned in 2018, so the material survided the incident; Dr. Adriano B. Kury);

**CAVAISC** Coleção de Artrópodes Vetores Ápteros de Importância em Saúde das Comunidades FIOCRUZ, Rio de Janeiro, Brazil (Dr. Marinete Amorim);

**MNHN**Muséum national d’Histoire naturelle, Paris, France (Dr. Mark Judson);

**CAS**California Academy of Sciences, San Francisco, USA (Dr. Darell Ubick).

## Results

### Taxonomy

#### Thelyphonidae Lucas, 1835

##### Typopeltinae Rowland & Cook, 1973

###### *Typopeltis* Pocock, 1894

**Type species**:

*Typopeltis*: *Typopeltiscrucifer* Pocock, 1894, by original designation.

*Gipopeltis*: *Typopeltisharmandi* Kraepelin, 1900, by original designation.

*Teltus*: *Teltusvanoorti* Speijer, 1936, by monotypy.

**List of *Typopeltis* species**:

1 – *T.amurensis* (Tarnani, 1889) (Russia), *nomen dubium*; 2 – *T.cantonensis* Speijer, 1936 (China); 3 – *T.crucifer* Pocock, 1894 (Japan, Taiwan); 4 – *T.dalyi* Pocock, 1900 (Thailand); 5 – *T.guangxiensis* Haupt & Song, 1996 (China); 6 – *T.harmandi* Kraepelin, 1900 (Vietnam); 7 – *T.kasnakowi* Tarnani, 1900 (Thailand); 8 – *T.magnificus* Haupt, 2004 (Laos); 9 – *T.sinensis* (Butler, 1872) (China); 10 – *T.soidaoensis* Haupt, 1996 (Thailand, Vietnam); 11 – *T.stimpsonii* (Wood, 1862) (Japan); 12 – *T.tarnanii* Pocock, 1902 (Thailand); 13 – *T.vanoorti* (Speijer, 1936) (China); 14 – *T.laurentianus* sp. n. (Vietnam).

####### Typopeltis laurentianus sp. n.

Taxon classificationAnimaliaUropygiThelyphonidae

http://zoobank.org/BE3AADFF-51A5-4F27-B039-346F0A280271

######## Type material.

***Holotype*** male: VIETNAM: Hà Tĩnh, 18.355240, 105.886949, 1998 (MNRJ 08243). ***Paratypes***: VIETNAM: Hà Tĩnh, 18.355240, 105.886949, 1998 (2 males, MNRJ 08243); Hà Tĩnh, 18.355240, 105.886949, 1997 (1 male, 1 female, MNRJ 08242); Quang Binh: Phong Nha-Kẻ Bàng National Park, 17.590802, 106.283344, 2001 (1 male, MNHN AR-UR-2 [ex MNRJ 08244]; 1 female, CAS, CASENT 9081667 [ex MNRJ 08245]); Vĩnh Phúc, 17.590802, 106.283344, x.1980, leg. R. Boistel (2 females, MNRJ 08246).

######## Etymology.

Species name *laurentianus* (laurentiana, laurentianum) is a Latin adjective after our friend, the distinguished Franco-Brazilian arachnologist Wilson Lourenço. The Latin form of Portuguese Lourenço is Laurentius (genitive Laurentiī), a noun of the second declension, cognate of English Lawrence or French Laurent. The ICZN allows authors of new species to choose the Latin version of contemporary names derived from Latin, which may be more euphonic than the modern counterparts.

######## Diagnosis.

Males (about 35 mm in total length without flagellum) larger than females (see measurements); males with patellar apophysis very long, with narrow base and apex, broader in the middle, with a small antero-posterior curve. Patellar apophysis without spines in the trunk or the terminal portion, with a smooth integument texture that differs from all the other species of *Typopeltis*. The male gonopod is simple, delimited by a sclerotized curved cuticle (posterior apex of **Fi**), with inverted trapezoid shape with rounded edges. The female gonopod has a bulbous **RS** with a wide base and a well marked **CCh**.

######## Description.

(Holotype male) ***Colouration (in alcohol).*** Reddish-brown. Carapace darker on anterior region than posterior region. Abdomen slightly yellowish. Pedipalps dark red, lighter in females lighter. Median eyes dark, almost black, lateral eyes yellow.

**Figure 1.**
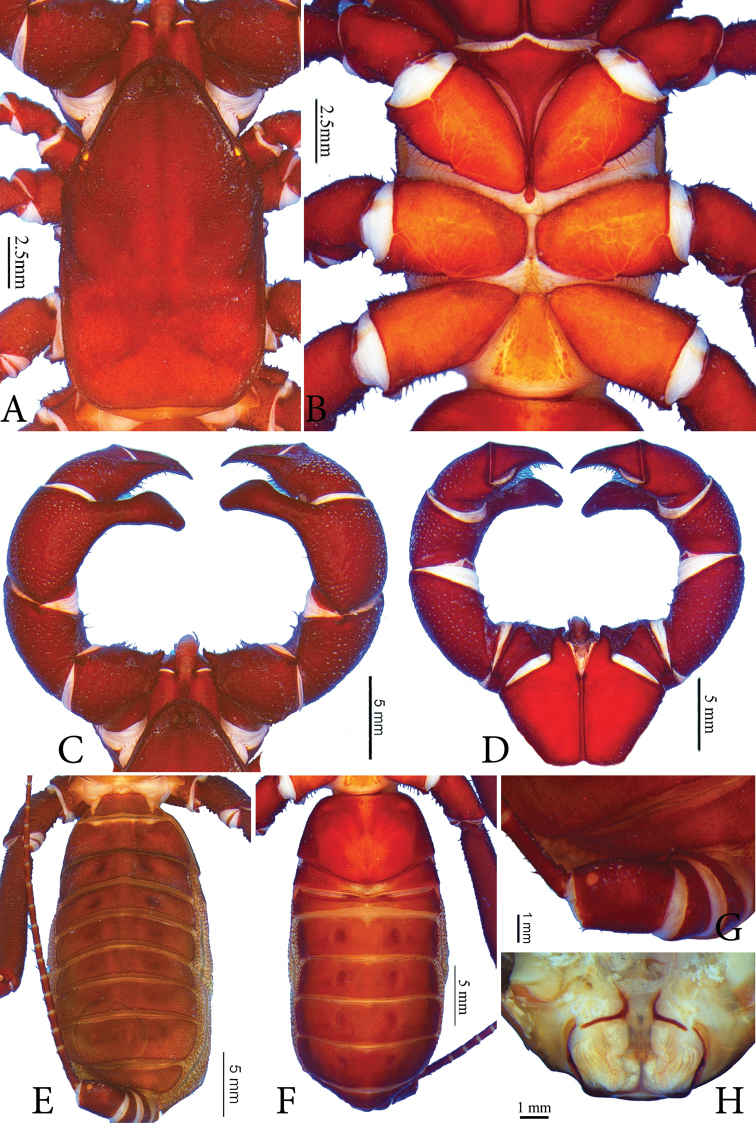
*Typopeltislaurentianus* sp. n., holotype (male). **A** Carapace **B** sternum **C** pedipalps (dorsal) **D** pedipalps (ventral) **E** opisthosoma (dorsal) **F** opisthosoma (ventral) **G** ommatoid **H** gonopod.

**Figure 2.**
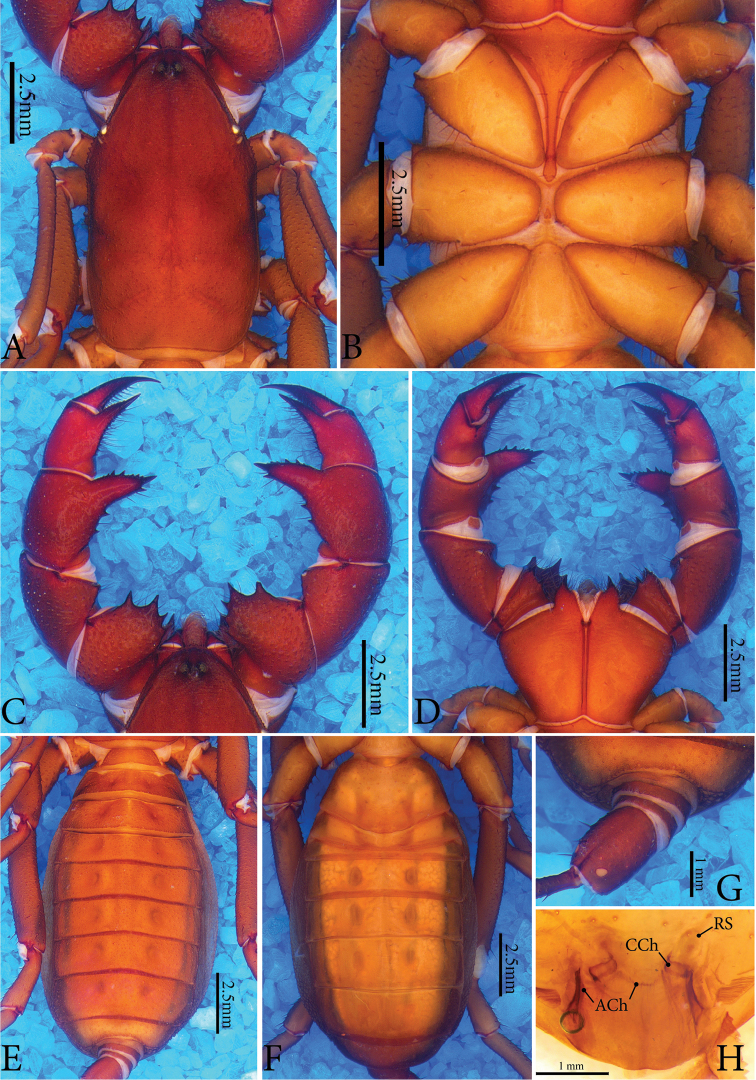
*Typopeltislaurentianus* sp. n., paratype (female). **A** Carapace **B** sternum **C** pedipalps (dorsal) **D** pedipalps (ventral) **E** opisthosoma (dorsal) **F** opisthosoma (ventral) **G** ommatoid **H** gonopod. **CCh** = circulus chitinosus; **ACh** = arcus chitinosus.

**Figure 3.**
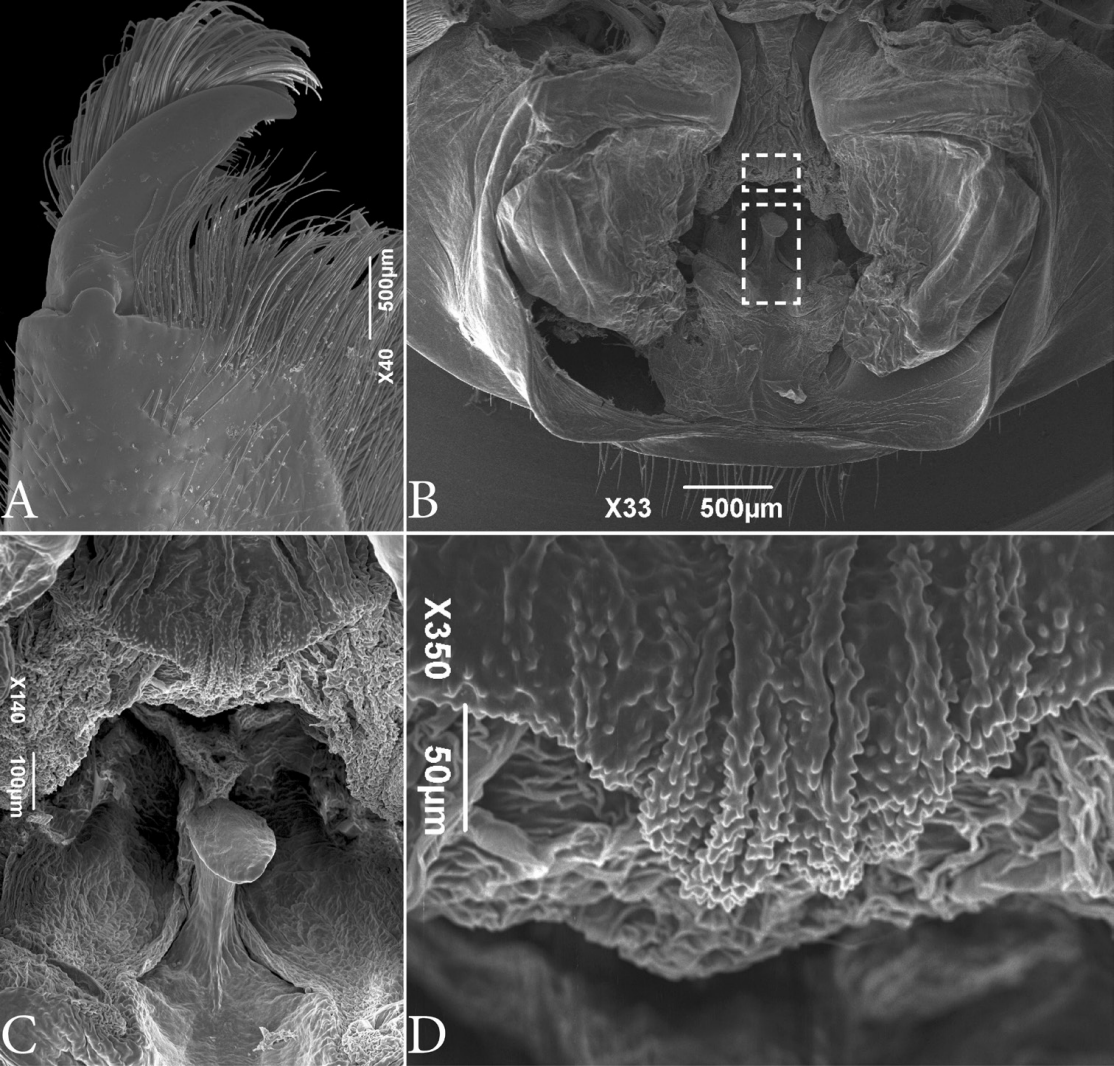
SEM images of *Typopeltislaurentianus* sp. n., paratype (male). **A** Chelicerae (right) **B** gonopod. Details in dashed rectangles are shown in images **C** (lower rectangle) and **D** (upper rectangle). **C** Fulcrum (**Fu**) detail **D** Mensa (**Me**) detail.

***Carapace*** (Figs 1A, 2A). With thick granules of irregular shapes homogeneously covering whole surface, granules interspaced. Lateral keel with one seta on each anterior end, next to median eyes; posterior end of keels above lateral triad of eyes, keel extends from posterior to anterior region of carapace; keels divided by median ocular ridge. Carapace has depression extending from posterior region of median ocular tubercle to region above subtriangular fovea. Median eye tubercle elevated, with well-marked ridge between eyes. Chelicerae with several setae in ventral region and on cheliceral claw. Cheliceral claw curved inwards, with thick base and narrow apex, and with short keel, smaller than half length of tooth (Fig. 3A).

***Sternum*** (Figs 1B, 2B). Typical of order, tri-segmented; inconspicuous mesosternum.

***Opisthosoma*** (Figs 1E, G; 2E, G). Pleura divided by crest of granules from tergites I–VIII; tergites without suture. Sides with fine granules (Figs 1E, 2E). Subcircular ommatoids present (Figs 1G, 2G). Flagellum with 38 articles (female paratype) and 36 in holotype (broken).

***Pedipalps*** (Figs 1C, D; 2C, D). **Coxa** without accessory tooth, with few setae. **Trochanter** punctated with granules covering dorsal surface. Four spines in dorso-mesal region (I <II <III <IV), spines I-III as broad as or broader than long, conical, with broad base and acute apex; spine IV geminate with spine III, with long setae, conical; apex rhomboid and bigger than double size of spine III. Two small spines close to articular condyle (trochanter-femur), spines smaller than mesal spine I (Fig. 1C). Ventral region with thick ridge all along joint with femur, ending mesally with two small conical spines, broader than long (Fig. 1D). **Femur** unarmed and covered with shallow pores concentrated on outer margin (Fig. 1D). Ventro-mesal region with reduced rhombus spine (almost a granule), conical, broader than long, surrounded by long setae (Fig. 1C). In females, ventro-mesal spine well developed, twice longer than wide, with very sharp, curved tip and broad base. Two small conical spines dorsally (I < II); in males these spines reduced to two small granules, clearly homologous to spines present in females. **Patella** covered by pores, especially on ectal face, with few setae; several setae mesally. Patellar apophysis almost as long as patella, with large non-terminal (median) expansion on external margin of apophysis (like large hump); unprecedented smooth texture and slight curvature in ventral direction on terminal portion. Apophysis with spatulated shape with slight concavity ventrally. Ventral face without spines. Females with two conical spines of subequal size in dorso-mesal view (Fig. 2C); spines as broad as long, with broad base and sharp tip, most anterior at base of apophysis. Ventral apophysis with reduced ventro-mesal spine in distal position (Fig. 2D). Patellar apophysis well developed, but slightly smaller than length of patella, conical, tapering towards apex; with single spine on mesal surface, positioned just before apex; row of spines with three or four small subequal basal spines on ectal face, followed by median series of four spines increasing in size; second series of spines larger than double first row of spines; distal series composed of three spines, with middle ones larger than two others. Two rows of setae at edges of ventral region, absent in males. **Tibia** covered by pores, large concentration of setae (in mesal view), more than in femur and patella. Tibial apophysis conically-shaped, broad base, acute apex, with series of dorsal spines. In ventro-mesal view with two small spines, most apical rhombic and double the size of previous one; penultimate spine with conical shape, with wide base. **Tarsus** covered by long setae, with greater predominance on mesal surface. With longitudinal series of ventral rhomboid spines and another dorsal series.

***Leg I.*** Eight tarsomers (variation: seven to nine), first very short (like small ring), second, third and last larger than others (I <II> III–VII <VIII); size and number of tarsomers can vary if leg is regenerated. Apical portion of tibia with two dorsolateral tricobothria, absent in femur and patella. Femur covered with thick granules, patella and tibia with smooth appearance. All articles covered with setae dorsally and ventrally.

***Legs II–IV.*** Trochanter and femur with granules. Coxa, tibia and tarsus smooth, last two with concentration of setae. With dorso-apical tricobothrium on tibia; ventro-apical region with thin, acuminate spur. Basitarsus with two spurs, one mesal and other ectal; ventral region with two longitudinal rows with four or five spiniform setae. Distitarsus divided into three tarsomers (I> II <III), length of tarsomere I equal or greater than II + III. Tarsomere I with two longitudinal rows with eight spiniform setae. Tarsomeres II and III similar to previous, but with three and four setae, respectively.

***Sternite*** (Figs 1F, 2F). Genital plate about 1.5 times wider than long, with irregularly distributed setae and accumulated pores on sides. Other ventrites mostly smooth.

***Male Gonopod*** (Figs 1H; 3B, C, 3D). **LoL1** broader than long, reniform, with thin longitudinal sclerotized wrinkles, slightly curved and sinuous in terminal portion (Fig. 1H); **Fi** with sclerotized borders and inverted trapezoid shape with rounded edges. **LoD** with strongly sclerotized acute projection positioned above all other gonopod structures. **LoL2** globose, soft, partially covered by **LoL1**; **LaM** as two parallel plates originating in **Me** and supported by **Fu** (Fig. 3C). **Me** subtriangular and covered by denticles (Fig. 3D). ***Female Gonopod*** (Fig. 2H) with seminal receptacle (**RS**) of bulbous shape, with base slightly narrower than more dilated distal portion; longer than wide; concave chitinous arc with two sclerotized chitinous rings at base of **RS**. Two well-sclerotized structures on sides of chitinous arch, very long and thin, slightly curved inwards, with base wider than apex.

######## Measurements.

(holotype male before brackets, variation inside brackets).

Prosoma: 13.6 mm (length) [12.0–13.6 mm], 8.0 mm (width) [7.1–8.0 mm]; Opisthosoma: 19.4 mm (length) [17.5–19.4mm], 10.7 mm (width) [8.3–10.7mm]; **Pedipalp**: Trochanter: 4.2 mm [3.7–4.2 mm]; Femur: 4.0 mm [4.0–4.4mm]; Patella: 5.4 mm [4.8–5.4 mm]; Patellar apophysis: 4.4 mm [4.0–4.4 mm]; Tibia: 4.1 mm [3.0–4.1 mm]; Tibial apophysis: 1.7 mm [1.6–1.8 mm]; Tarsus: 3.1 mm [2.6–3.1 mm].

####### Typopeltis guangxiensis

Taxon classificationAnimaliaUropygiThelyphonidae

Haupt & Song, 1996

######## Studied material.

CHINA: Guangxi: Nanning: Gao Feng Park, 22.955023, 108.365636, 136 m, 13.vii.2016, leg. A. Giupponi, A. Kury, I. Kury & C. Zhang (3 females, MNRJ 08249); same locality, 13.vii.2016, leg. A. Giupponi, A. Kury, I. Kury & C. Zhang (3 juvenile males, 2 juvenile females, MNRJ 08250). Fangshenggang: Shi Wan Danshan National Park, 21.90538, 107.90366, 276 m, 11–12.vii.2016, leg. A. Giupponi, A. Kury, I. Kury & C. Zhang (1 male, MNRJ 08251); same locality, 11–12.vii.2016, leg. A. Giupponi, A. Kury, I. Kury & C. Zhang (2 juvenile males, MNRJ 08252).

######## Emended diagnosis (after Haupt 1996).

Males (about 30 mm in total length without flagellum and chelicerae) larger than females (see measurements); very long patellar apophysis with narrow base and almost straight, blunt tip with three small blunt terminal projections. Male gonopod trapezoidal, **LoL1** reniform with longitudinal sclerotized streaks, **Me** square, covered by denticles, with four longitudinal crests partially formed by collapsed spines (observable only in SEM). Female gonopod with bulbar **RS** with wide base and well-marked **CCh** with large bevel in upper inner portion; **ACh** concave.

######## Description.

***Colouration (in alcohol).*** Male blackish red, carapace colour becoming lighter from anterior to posterior region. Pedipalps darker in relation to body; legs II–IV lighter compared to carapace. Middle eyes black; lateral eyes yellow. Females slightly lighter than males, more reddish in general.

**Figure 4.**
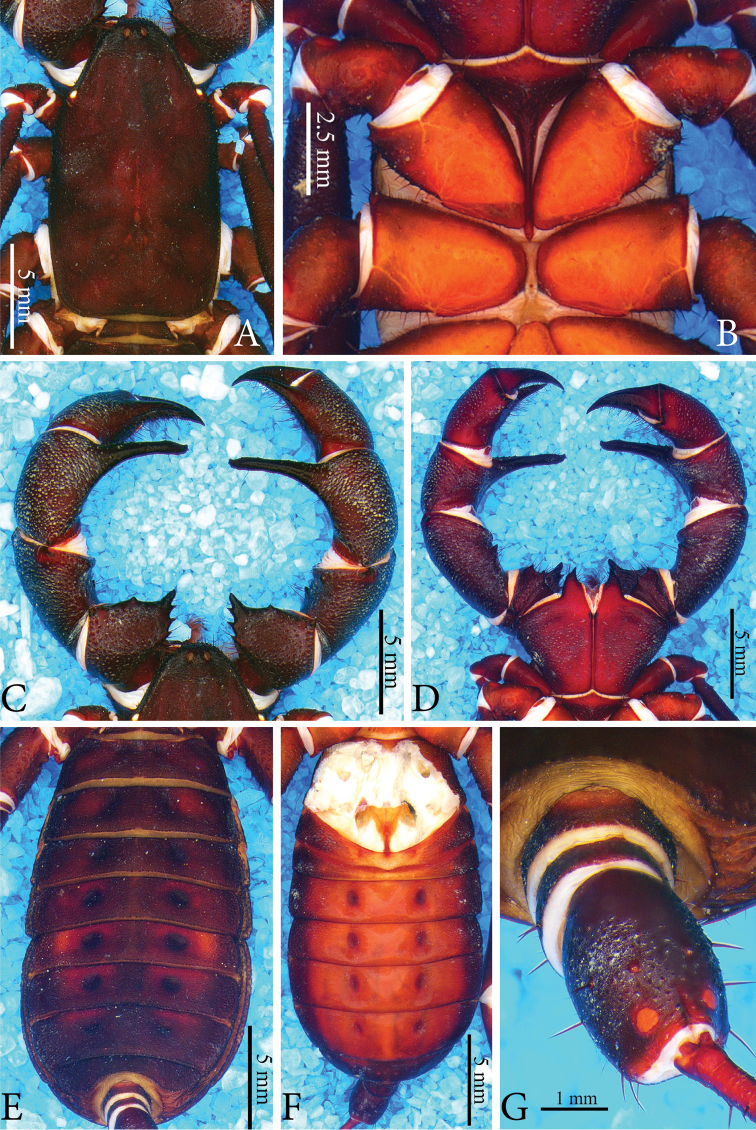
*Typopeltisguangxiensis* (male). **A** Carapace **B** sternum **C** pedipalps (dorsal) **D** pedipalps (ventral) **E** opisthosoma (dorsal) **F** opisthosoma (ventral) **G** ommatoids.

**Figure 5.**
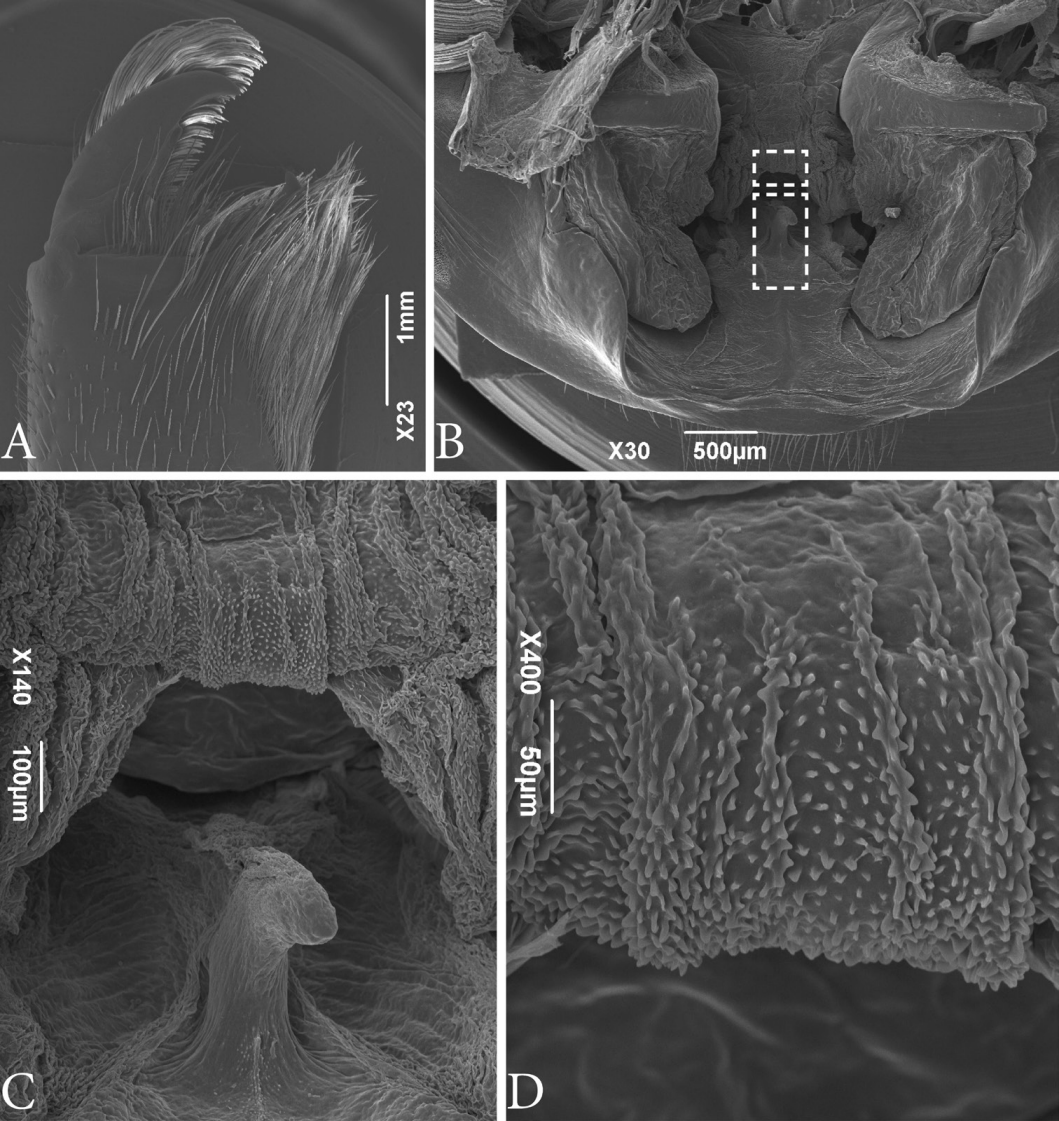
SEM images of *Typopeltisguangxiensis* (male). **A** Chelicerae (right) **B** gonopod. Details in dashed rectangles are shown in images **C** (lower rectangle) and **D** (upper rectangle). **C** Fulcrum (**Fu**) detail **D** Mensa (**Me**) detail.

***Carapace*** (Fig. 4A). Granules without specific pattern. Keel present between median and lateral eyes. Deep line flanked by granules from posterior region of median eyes to slightly above fovea. Chelicerae similar to *T.laurentianus*, but setae apparently thinner and denser ventrally. Chelicerae claw curved inwards, with thick base and narrow apex, with keel longer than half-length of chelicerae claw (Fig. 5A).

***Sternum*** (Fig. 4B). Typical tri-segmented sternum; inconspicuous mesosternum.

***Tergites*** (Fig. 4E, G). Acute granules present at posterior border of each tergite, absent in small central region of posterior border of tergites I–V. Ommatoids subcircular.

***Pedipalps*** (Fig. 4C, D). **Coxa** covered in setae, with higher concentration in ventro-apical and latero-apical portions. **Trochanter** dorsally armed with 5 spines; spines I, II, III increasing in size, facing inwards; spine IV more than twice larger than others, rhomboid and paired with spine III in apical position; spine V smallest, rhombus (I <II <III <IV> V) (Fig. 4C). Two short, broad-based subequal spines ventrally (Fig. 4D). **Femur** dorsal face covered in thick granules, with few ectal setae and single reduced mesal spine (Fig. 4C). One ectal-ventral spine much larger than dorsal one (about four times) (Fig. 4D). **Patella** dorsal face with many pores with no apparent pattern, patellar apophysis slender, with small spines on anterior face, enlarged apex with three globular expansions (Fig. 4C). Ventral face without spines (Fig. 4D). **Tibia** dorsally armed with large conical apophysis with sharp tip, slightly smaller than tibia, covered in setae, with two longitudinal series of small spines, dorsal series with eight small spines, ventral series with fourteen spines (Fig. 4C). Ventrally armed with two ectal conical spines, distal almost double the size of subdistal (Fig. 4D). **Tarsus** armed dorsally with longitudinal series of fourteen small spines (Fig. 4C), armed ventrally with ten spines (Fig. 4D).

***Leg I (antenniform)*** with nine tarsomers, first very short (as small ring); second, third and last tarsomers longer than others. Number of tarsomeres may vary if there is regeneration of tarsomeres. Apical portion of tibia with two dorsolateral trichobothria, absent on femur and patella. Femur covered with thick granules, patella and tibia with smooth appearance. All articles covered with setae, dorsally and ventrally. ***Legs II–IV.*** Trochanter and femur with granules. Coxa, tibia and tarsus smooth, last two with concentrated setae. Dorsal-apical trichobothrium present on tibia, thin acuminate spur on ventro-apical region of tibia. Basitarsus with two spurs, one mesal and one ectal; ventral region with two longitudinal rows of five spiniform setae. Distitarsus divided into three tarsomers (I> II <III), I equal or greater than II + III. Tarsomere I with two longitudinal rows of nine to ten spininiform setae each. Tarsomeres II and III with similar structure, but with three and four setae, respectively.

***Sternites*** (Fig. 4F). Genital plate wider than long (one and a half times wider than long). Sternite mostly smooth, with granules concentrated on sides. Sternite II with large number of setae and central acute granule on posterior margin. Flagellum with thirty-eight articles.

**Figure 6.**
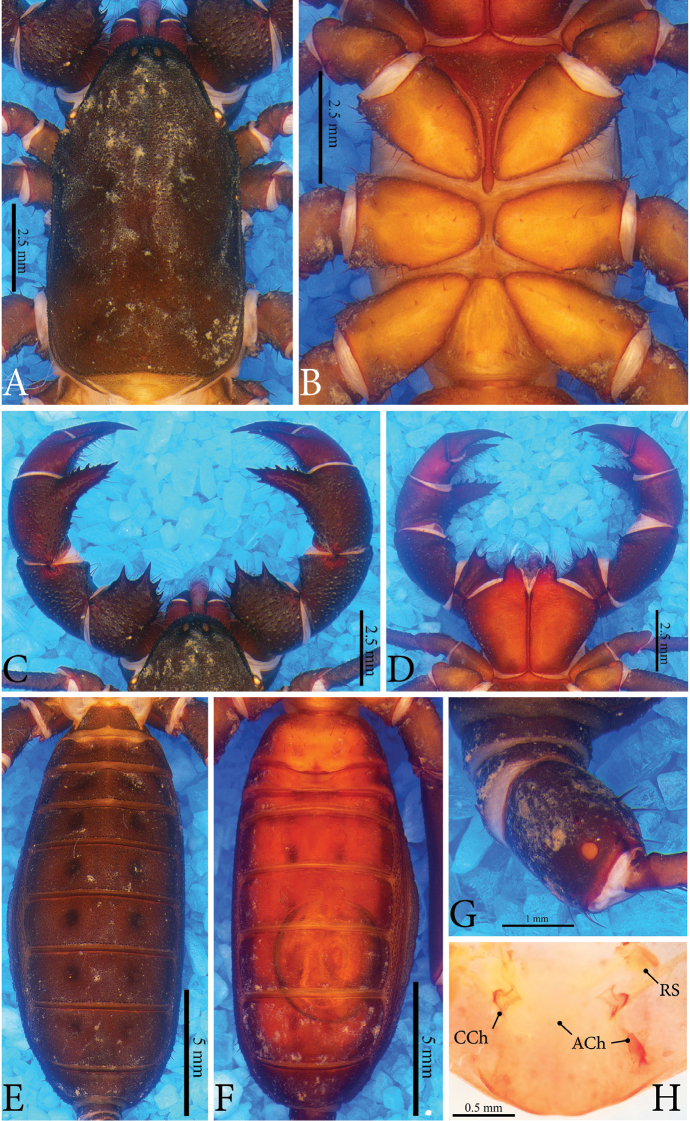
*Typopeltisguangxiensis* (female). **A** Carapace **B** sternum **C** pedipalps (dorsal) **D** pedipalps (ventral) **E** opisthosoma (dorsal) **F** opisthosoma (ventral) **G** ommatoids **H** gonopod. **CCh** = circulus chitinosus; **ACh** = arcus chitinosus.

***Male gonopod*** (Fig. 5B, C, D). **LoL1** broader than long, reniform, with thin longitudinally sclerotized striations, slightly curved and sinuous in terminal portion; **Fi** with sclerotized borders, with inverted trapezoid shape with rounded edges. In basal portion, **Fi** and **LoD** are not fully fused. **LoL2** globose and partially covered by **LoL1**; **LaM** as two parallel plates, originating in **Me** and supported by **Fu** (Fig. 5C). **Me** square, covered by denticles, with four longitudinal crests formed partially by collapsed spines (Fig. 5D). ***Female gonopod*** (Fig. 6H), **RS** of bulbous shape, with base narrower than more dilated portion, not much longer than wide; **ACh** chitinous, concave, with two **CCh** sclerotized at base of **RS**, with large chamber in upper inner portion. On sides of **Ach**, the two long and thin sclerotized structures (observed in *T.laurentianus* sp. n.) are absent.

######## Natural history.

Collected on the ground of forested areas in the outskirts of Nanning (Guangxi, China), living under logs and stones in shady and humid places. The specimens were abundant in habitats associated with human disturbance, such as roadsides, trails, and abandoned constructions in the forest.

######## Measurements.

(male)

Prosoma (length): 14.0 mm; Prosoma (width): 8.0 mm; Opisthosoma (length): 16.8 mm; Opisthosoma (width): 10.3 mm. **Pedipalp**- Trochanter: 4.3 mm; Femur: 3.5 mm; Patella: 5.6 mm; Patellar apophysis: 4.2 mm; Tibia: 4.0 mm; Tibial apophysis: 2.1 mm; Tarsus: 3.8 mm. **Genital Plate**- Length: 6.0 mm; Width: 9.0 mm.

## Discussion

**Figure 7.**
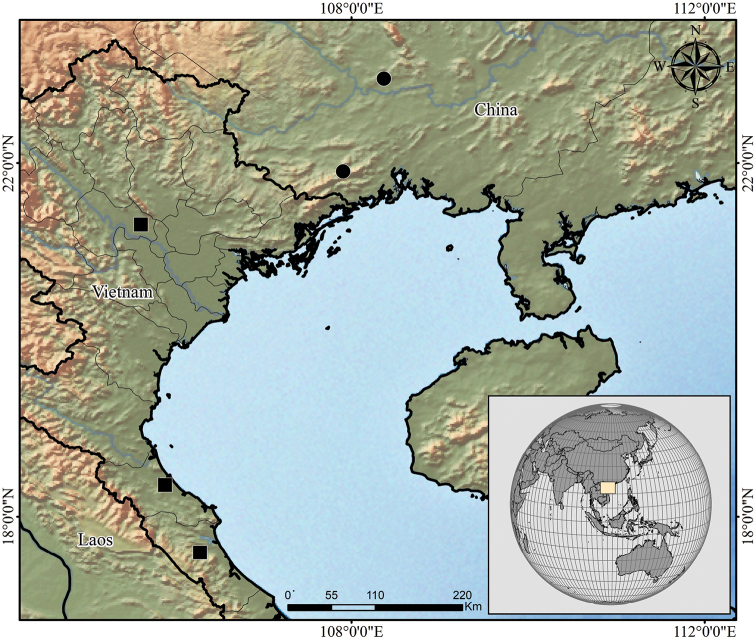
Distribution map of *Typopeltislaurentianus* sp. n. (black squares) and *T.guangxiensis* (black circles).

The taxonomy of Southeast Asian whip scorpions was greatly advanced by Haupt (1996, 2004a, b, 2009) and Haupt and Song (1996). However, due to the large geographical extension of the region, a lot of work still needs to be done to fully comprehend the diversity of thelyphonids in the area. The genus *Typopeltis* alone has a large distribution covering China, Japan, Laos, Russia, Taiwan, Thailand and Vietnam. Currently, only 14 species are known in the genus. *Typopeltislaurentianus* sp. n. is the third species of the genus described from Vietnam (Fig. 7). The others are *T.harmandi*, known only from females from the southern tip of the country, and *T.soidaoensis*, which has also been recorded from Thailand.

*Typopeltislaurentianus* sp. n. differs from the others in the genus by the unique shape of the patellar apophysis of the male. In *T.soidaoensis* the apophysis is thinner, tapering uniformly, with a dorso-ventral curvature and small apical digitiform structures (see Haupt 1996: fig. 1d), whereas in the species from southern China, *T.guangxiensis* and *T.cantonensis*, the apophysis is straight, thin and has a blunt tip armed with apical denticles (Fig. 4C, D for *T.guangxiensis*; see Haupt and Song 1996: fig. 2d for *T.cantonensis*). In *T.magnificus*, which occurs on the Laos border, the apophysis is similar to that of *T.soidaoensis*, but it is thinner and longer. Females in general have thinner patellar apophyses than males (see Haupt 2004: figs 1, 3).

*Typopeltis* commonly have a notable expansion in the terminal region of the patellar apophysis of the male pedipalps (secondary sexual dimorphism), which is generally armed with spines, digitiform projections or large granules (see *T.crucifer*, *T.dalyi*, *T.niger*, *T.stimpsonii*, and *T.tarnanii*) (Haupt 1996, Haupt and Song 1996). The other species, despite having some type of apical structure in the patellar apophysis of males, do not have the pronounced expansion. *Typopeltislaurentianus* sp. n., on the other hand, has a great median expansion, located only on the external margin of the apophysis, like a large hump, in addition to having an unprecedented smooth texture. The apophysis presents a slight curvature in the final portion in the ventral direction (Fig. 1C, D), as was observed in *T.kasnakowi* and *T.vanoorti* (Haupt 1996, Haupt and Song 1996), where only the terminal region is curved. *Tylopeltisstimpsonii*, *T.soidaoensis*, *T.magnificus* and *T.tarnanii* differ from the new species by having a more pronounced ventral curvature that is not restricted to the terminal portion. According to Haupt (1996) and Haupt and Song (1996), *T.crucifer* has the curve facing the tibia, while *T.cantonensis* has a straight apophysis, characteristic also for *T.guangxiensis* (Fig. 4C, D). The species *T.amurensis*, *T.harmandi* and *T.kasnakowi* are only known from female specimens.

The keel of the carapace in *T.laurentianus* sp. n. does not reach the front of the middle eyes (Fig. 1A), a character described in Haupt (1996) and Haupt and Song (1996) for most species of *Typopeltis*, with the exception of *T.dalyi*, *T.crucifer*, *T.niger* and *T.stimpsonii*. In *T.magnificus*, Haupt (2004) does not comment on whether or not the keel reaches the median eye tubercle, but the illustration shows that it does not reach.

**Figure 8.**
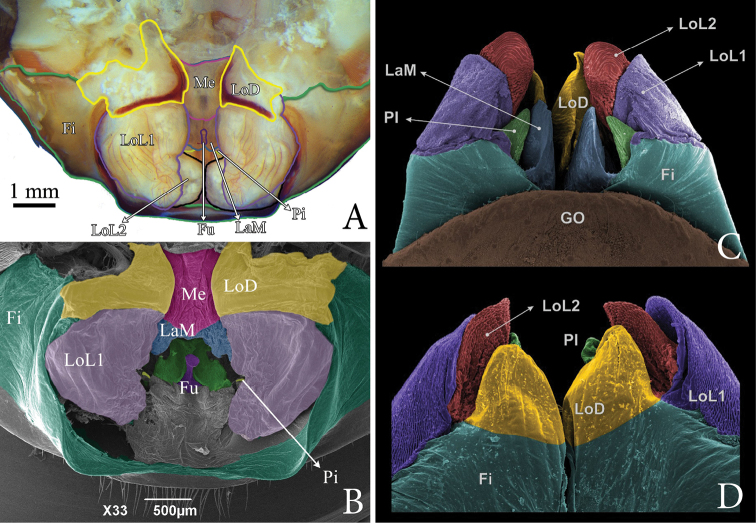
Comparison between gonopods of *T.laurentianus* sp. n. (**A, B**) and *Heterophrynus* sp. Pocock, 1894 (**C, D**). **A** Male gonopod of *T.laurentianus* in posterior-dorsal view **B**SEM of male gonopod of *T.laurentianus* in posterior-dorsal view **C**SEM of male gonopod of *Heterophrynus* sp. in ventral view **D**SEM of male gonopod of *Heterophrynus* sp. in dorsal view. **Fi** = fistula; **GO** = genital operculum; **LaM** = lamina medialis; **LoD** = lobus dorsalis; **LoL1** = lobus lateralis primus; **LoL2** = lobus lateralis secundus; **PI** = processus internus; **Me** = Mensa; **Fu** = Fulcrum.

Male gonopods in Pedipalpi (Schizomida, Thelyphonida and Amblypygi) are formed by soft structures used to shape the extruded spermatophore. Those structures were studied in detail in amblypygids by Giupponi and Kury (2013), but are barely known in the other groups. Here we presented details of the male gonopod of thelyphonids for the first time with SEM images. This allowed us tentative homologies of the male genitalic structures between the two orders based on their position and shape (Fig. 8). The most external and fleshy tubes with a smooth surface are recognized as being a homologue of the *fistula* (**Fi**) of amblypygids. In thelyphonids, the distalmost apex of the *fistula* is chitinized and projects posteriorly. Dorsal to the **Fi** is a pair of chitinized projections, the *Lobus dorsalis* (**LoD**), that cover the other gonopod structures. Different from amblypygids, the **LoD** in Thelyphonida does not form a tube and does not cover the gonopod completely dorsally, thus allowing us to see the other parts of the gonopod. On the tip of the **Fi** two telescoping soft bodies are projected; they are the *Lobuslateralisprimus* (**LoL1**), which has sclerotized wrinkles in its surface. Beneath **LoL1**, a pair of smooth soft bodies are present, the *Lobuslateralissecundus* (**LoL2**). Inner to **LoL2**, there is a soft blade from the *fistula*’s median-dorsal part, which is preceded by a leaf-like lamina, the *Lamina medialis* (**LaM**). Male gonopods of whip scorpions have structures unique to the group, such as the *Fulcrum* (**Fu**) and *Mensa* (**Me**). The **Fu** is sclerotized and seems to be a supporting structure. The **Me** covers the upper part of the **LaM**.

As far as we know, there are no published images of male gonopods of *Typopeltis*. Haupt (2009) published images of the gonopod of other genera of Thelyphonida and studied the structure superficially; in that work the sclerotized region here named LoDwas called “cuticular clasp”. We prefer not to use the same name because the chitinized region appears to be stationary, and therefore does not work as a clasp. Additionally, no gonopod characters were used for taxonomy by Haupt (2009). In the SEM images of the gonopod illustrated here it is possible to identify ultrastructures not observable in traditional microscopy (Figs 3B, C, D; 5B, C, D; 8), such as the **Fu** and **Me**. While **Fu** seems to be a supporting structure of the **LaM** (which in the SEM images is collapsed, thus allowing the **Fu** to be observed), apparently it does not show differences between the two studied species. In the case of **Me**, there is a noticeable difference in integument texture and shape between the two species. In *T.guangxiensis* the general form of **Me** is similar to a square with four crests of rhombus spines separated by three areas of lamellar spines. In *T.laurentianus* sp. n. **Me** is subtriangular with more pronounced ridges, blunt, and often with paired teeth.

Females of *Typopeltis* do not have secondary sexual characters and are more homogeneous morphologically than the males. The identification of species based only on females is, therefore, more difficult. Additionally, informative diagnostic structures (such as the gonopod) are rarely depicted in scientific papers, making it even harder to use females to separate species. Only the female gonopods of *T.guangxiensis* and *T.crucifer* are known in the literature (Haupt and Song 1996, Haupt 2009). In some cases, it is possible to separate the females by evident characters of external morphology, such as the spines of the patellar apophysis on the pedipalps. In *T.laurentianus* sp. n. the mesal face has a row of spines with a distinct size relation (see description); on the ectal face, it has only one spine in the final third, besides the second dorsal-patellar spine that is practically at the base of the apophysis (Fig. 2C, D). *Typopeltisguangxiensis* (Fig. 6) has a particular size relation of spines on the mesal part of the patella; additionally, besides the two usual spines, females have two smaller (smaller than half of the other two) spines in the middle third (Fig. 6C, D). *Typopeltismagnificus* also has a specific size pattern in the mesal row; in the ectal face it has a third spine subequal to the others, in the medial third of the patellar apophysis.

## Supplementary Material

XML Treatment for Typopeltis laurentianus

XML Treatment for Typopeltis guangxiensis
